# Effects of EFNA1 on cell phenotype and prognosis of esophageal carcinoma

**DOI:** 10.1186/s12957-021-02362-8

**Published:** 2021-08-16

**Authors:** Yongqiang Zhang, Jinning Zhang, Guanlong Pan, Tianhao Guan, Changhao Zhang, An Hao, Yan Li, Hai Ren

**Affiliations:** Ward 2, Department of Cardiothoracic Surgery, The Third Affiliated Hospital of Qiqihar Medical College, No. 27, Taishun Street, Tiefeng District, Qiqihar, 161000 Heilongjiang Province China

**Keywords:** EFNA1, Esophageal carcinoma, Prognosis, Cell proliferation

## Abstract

**Background:**

To investigate the expression and clinical significance of EFNA1 in broad-spectrum tumors, and to evaluate its relationship with prognosis and biological functions of esophageal carcinoma (ESCA).

**Methods:**

EFNA1 expression in various cancers was analyzed according to the data in the TCGA database. The clinical data were integrated, to analyze the relationship with ESCA clinical parameters and prognosis, and EFNA1 expression in ESCA tissue samples was detected by immunohistochemistry (IHC). Based on bioinformatics, the functional background of EFNA1 overexpression was analyzed. EFNA1 knockout cell model was established by EFNA1-shRNA transfecting ESCA cells, and the effect of knocking down EFNA1 on the proliferation of ESCA cells was detected by MTT.

**Results:**

Among 7563 samples from TCGA, the EFNA1 gene highly expressed in 15 samples with common cancers and endangered the prognosis of patients with tumors. Its overexpression in ESCA and its influence on the prognosis were most significant. EFNA1 expression in 80 samples with ESCA and their paired samples was tested by IHC to verify its high expression (paired t test, *P* < 0.001) in ESCA tissues. It was found that EFNA1 expression was related to clinical factors (TNM staging, *P* = 0.031; lymph node metastasis, *P* = 0.043; infiltration, *P* = 0.016). Meanwhile, EFNA1 was found to be an independent risk factor based on the COX multi-factor analysis. And to further explore the importance of EFNA1 in tumors, EC-9706 and ECA109 cells were screened from 8 ESCA-related cell lines to build EFNA1 knockdown cell models. The results showed that EFNA1 knockdown significantly inhibited the proliferation of tumor cells (*P* < 0.05). In terms of molecular mechanism, EFNA1 related genes were significantly enriched in the proliferative pathway according to the pathway enrichment analysis. It was found that knocking down EFNA1 did inhibit cell proliferation based on cell experiments.

**Conclusions:**

EFNA1 overexpression in ESCA tissue is related to the prognosis of patients. Knocking down EFNA1 can significantly inhibit the proliferation of ESCA cells.

**Supplementary Information:**

The online version contains supplementary material available at 10.1186/s12957-021-02362-8.

## Background

Esophageal carcinoma (ESCA) is the eighth most common cancer and the sixth most common cause of cancer death in the world [1]. As one of the common malignant tumors, the number of new and dead cases of ESCA ranked 7th and 6th among the tumors according to the Global Cancer Society Report 2018 [2]. Currently, ESCA has high incidence in China, with higher mortality and morbidity than the global average. In China, the occurrence of ESCA is geographically clustered, which may be related to living conditions, dietary habits, and genetic susceptibility [3]. The early symptoms of ESCA are not typical. Progressive dysphagia is the main complaint of most patients with ESCA, which is often the advanced stage of the disease [4]. At present, surgery is the first choice for ESCA, but the tumor recurrence rate is high after surgery, which is easy to be distant metastasis, with poor long-term effect. It will also cause skeletal muscle loss and affect patients’ quality of life [5]. Therefore, it is of great significance to find biomarkers for early ESCA screening and to predict the targets for the prognosis of ESCA.

Eph (erythropoietin-producing hepatoma) receptor is the largest receptor tyrosine kinase (RTK), which can be divided into two subcategories according to sequence homology, including EphA1-10 and EphB1-6 [6]. EFNs (ephrins) is a ligand for Eph receptor which can be divided into two subcategories according to its connection with the membrane, among which, EFNAs (EFNA1-5) is fixed to the membrane by glycosyl-phosphatidyl inositol (GPI), while EFNBs (EFNB1-3) is immobilized through the transmembrane protein domain [7]. EFNA1 has about 30–40% similarity to other EFNs, which can bind to multiple EPHA receptors (EPHA1-5) [8]. Among which, EphA2 is the most common receptor of EFNA1. The binding of ligand EFNA1 to receptor EphA2 can promote phosphorylation, which in turn regulates cell growth, differentiation, apoptosis, and angiogenesis, so as to be involved in tumorigenesis and metastasis [9, 10]. Literature have documented that EFNA1 extensively involved in tumorigenesis by influencing tumor angiogenesis [11, 12], malignant cell events [13, 14], and invasiveness [15]. Its expression is up-regulated in many cancers (e.g., gastric cancer [16], colorectal cancer [17], renal cancer [18]), which is closely related to the prognosis of many cancers [19, 20]. The study by De Robertis et al. has also found that EFNA1 may become a prognostic marker for colorectal cancer [21]. Current studies have found EFNA1 to be associated with the prognosis and progression of many tumors, including digestive system tumors. However, EFNA1 expression in ESCA and its relationship with the prognosis of patients, as well as its role in tumor progression, remain unclear and need further study.

Therefore, this study first systematically analyzed EFNA1 in a variety of tumors and found that its high expression in ESCA is the most significant and is related to the prognosis in ESCA. We further explored the relationship between EFNA1 and the proliferation of ESCA cells at the cell level, providing basic theoretical basis for studying the pathogenesis of ESCA and prognosis evaluation of patients.

## Materials and methods

### Data mining

The mRNA expression spectrum and clinical data of patients with common tumors and controls were downloaded from the Cancer Genome Map TCGA (https://tcga-data.nci.nih.gov/tcga/) Database. Data were preprocessed by sorting, ID converting, filtering, merging, correcting the data from different expression spectrum and extracting clinical data. EFNA1 expression in each tumor was analyzed with the “meta” software package of R software (version 3.6.3), and the influence of EFNA1 expression on the prognosis of each tumor was analyzed with the “survival” software package of R software. Through univariate COX analysis, the clinical data and EFNA1 expression were analyzed, as well as the scanned parameters with *P* < 0.02. In order to predict the potential functions of EFNA1, the differentially expressed genes (DEGs) of patients with ESCA were identifies in the TCGA data with the edgeR package in the R software that were divided into high expression group and low expression group. |log2FC| > 1 and calibration *P* < 0.05 were taken as cutoff values for DEGs screening. After that, DEGs were analyzed with GO terms to predict their functions. The pathway with *P* < 0.05 was considered statistically significant. STRING revealed that the interaction between these DEGs was visualized by the protein interaction (PPI) network.

### Clinical samples

The pathological specimens surgically removed from patients in the Department of Cardiothoracic Surgery, the Third Affiliated Hospital of Qiqihar Medical University, from December 2014 to December 2020, were selected as specimens for the remaining paraffin sections after pathological examination. Inclusion criteria are as follows: (1) patients undergoing surgical resection in the Thoracic Surgery Department of our hospital, (2) patients with pathology diagnosed as ESCA, and (3) patients with primary tumor foci. Exclusion criteria are follows: (1) patients who have received preoperative radiotherapy and chemotherapy, (2) patients received second operation, and (3) patients with severe cardiopulmonary and other basic diseases. The adjacent tissues were taken 2–3 cm away from the tumor and histopathologically confirmed to be non-cancerous tissues. Each specimen was stored in liquid nitrogen 15 min in vitro for subsequent experiments. This study has been approved by the Ethics Committee in our hospital.

### Immunohistochemical (IHC) and pathological score

Paraffin sections were baked at 60 °C for 2 h, dewaxed with xylene (Shanghai Sangon, China), as well as gradient ethanol dehydration, and were washed by PBS, which were then repaired with high pressure antigen for 10 min, and washed PBS again. The sections were soaked in 3% H_2_O_2_ for 10 min to block exogenous peroxidase activity, closed with goat serum for 10 min (at room temperature), and then were incubated with added rabbit anti-human EFNA1 antibody (ab238505, Abcam, USA, 1:100) at 4 °C overnight. The above sections were incubated with sheep anti-rabbit II (6926-100, Emmett Technology, China, 1:1000) at room temperature for 20 min and colored with added DAB (Zhongshan Jinqiao, Beijing) for 3 min. Hematoxylin (Shanghai Sangon, China) was added for re-dying for 2 min, with gradient ethanol for dehydration and resinene for sealing, which was placed under the microscope (Olympus, Japan) for observation.

The immunopathology score was performed by 2 senior pathology physicians in our hospital, and the total result was given according to the total score of the product of dyeing area and intensity. Dyeing area: < 10% (0 point), 10–49% (1 point), 50–75% (2 points), > 75% (3 points); Dyeing intensity: uncolored (0 point), pale yellow (1 point), brown (2 points), dark brown (3 points), the total score ≥ 4 represents high expression, and the total score < 4 represents low expression.

### Cell culture

Human esophageal carcinoma cell lines (EC-9706, ECA109, KYSE140, KYSE510, TE-10, TE-1, KYSE70) and human normal esophageal epithelial cell line HEEC were purchased from the Cell Bank of the Chinese Academy of Sciences. The cell DMEM (Corning, USA) was cultured with culture medium, with 10% fetal bovine serum (Thermo, USA) and 1% penicillin (Corning, USA). Conditions of culture: The cells were cultured in a constant temperature incubator at 37 °C and 5% CO_2_.

### Real-time fluorescence qRT-PCR experiment

RNA was extracted with Trizol method (15596026, Invitrogen, USA), which was then retranscripted as cDNA with TaKaRa Mini BEST FFPE DNA Extraction kit (RR047A, TaKaRa, Japan). Sampling was performed with SYBR Premix EX Taq kit (RR420A, Takara), which was amplified with ABI Step One Plus TM reaction apparatus (USA). Taking GAPDH as the internal reference gene, reaction system: SYBR Mix 9 μL, forward primer 0.5 μL, reverse primer 0.5 μL, cDNA 2 μL, RNase Free dH2O 8 μL, reaction conditions: at 95 °C for 10 min, at 95 °C for 15 s, at 60 °C for 1 min, for 40 cycles. The 20 μL reaction system was taken for RT-PCR. The target gene (EFNA1) and the internal reference gene (GAPDH) were amplified under the same reaction conditions, the relative expression of target gene was analyzed with 2^−ΔΔCt^ (Table 1).

**Table 1:** Primer sequence list

Primer name	Primer sequence
EFNA1	5′-CCGCTCATCGTGCAACCTG -3′ (Forward)
	5′-ATAAGAAGGCATCAGATCG-3′ (Reverse)
GAPDH	5′-TCCTTCAGCTTCCACGAG-3′ (Forward)
	5′-TCCTTCAGCTTCCACGAG-3′ (Reverse)

### Western blot

The total cell protein was extracted, and then was detected with BCA method (Beyotime, China) for protein concentrations. After preparing the glue, the protein was isolated with SDS-PAGE electrophoresis. With transfer electrophoresis device, the protein was transferred to PVDF membrane (Millipore, USA) by wet transfer method at 4 °C, under constant current of 300 mA for 90 min, and sealed with TBST solution containing 5%BSA at room temperature for 1 h. Rabbit anti-human EFNA1 antibody (AB124911, abcam, 1:1000), rabbit anti-human CDK2 antibody (AB182858, abcam, 1:2000), rabbit anti-human cyclInd antibody (AB32503, abcam, 1:1000), and rabbit anti-human cyclIne antibody (AB13847, abcam, 1:500) were added for incubation at 4 °C overnight, which were reacted with corresponding secondary antibodies (sheep anti-rabbit IgG, SantaCruz, SC-2004, USA, 1:2000). The bands of antigen-antibody binding region were detected with chemiluminescence method and analyzed by ImageJ software.

### Cellular transfection

EFNA1-shRNA1 (5′CCGCGTCTTCACTGCAACG3′) and EFNA1-shRNA2 (5′CTAGCAGAGATGACAATG3′) were purchased from Genechem Company (Shanghai, China). Cells were inoculated on the 6-well plates (Corning, USA) and cultured to a fusion of 50–70%. According to the instructions of Lipofectamine® 3000 Transfection kit (Invitrogen, USA), 10 μL transfection reagent was diluted with 250 μL serum-free medium, added into each hole of culture plates, and incubated for 4 h for subsequent experiments. The transfected EFNA1-shRNA was grouped into the EFNA1-shRNA group, and the transfected blank plasmid was grouped into the negative control (NC) group.

### MTT detection

After transfecting for 36 h, the cells were inoculated in the 96-well plates at 5 × 10^3^ cells/hole. After inoculation for 1, 2, 3, 4, and 5 days, 20 μL MTT solution was added to each hole and incubated for 4 h, the OD value was measured at 490 nm wavelength, and the cell growth curve was drawn.

### Statistical analysis

The experiment was performed with SPSS 24.0 (SPSS, Chicago, USA) software for 3 times. Measurement data materials were processed with paired sample *t* test and ANOVA. Counting data were compared with *x*^2^ test. *α* = 0.05 was taken as the test standard, and *P* < 0.05 was defined as that the difference was statistically significant.

## Results

### EFNA1 expression in tumor tissues and its clinical significance

**Fig. 1:**
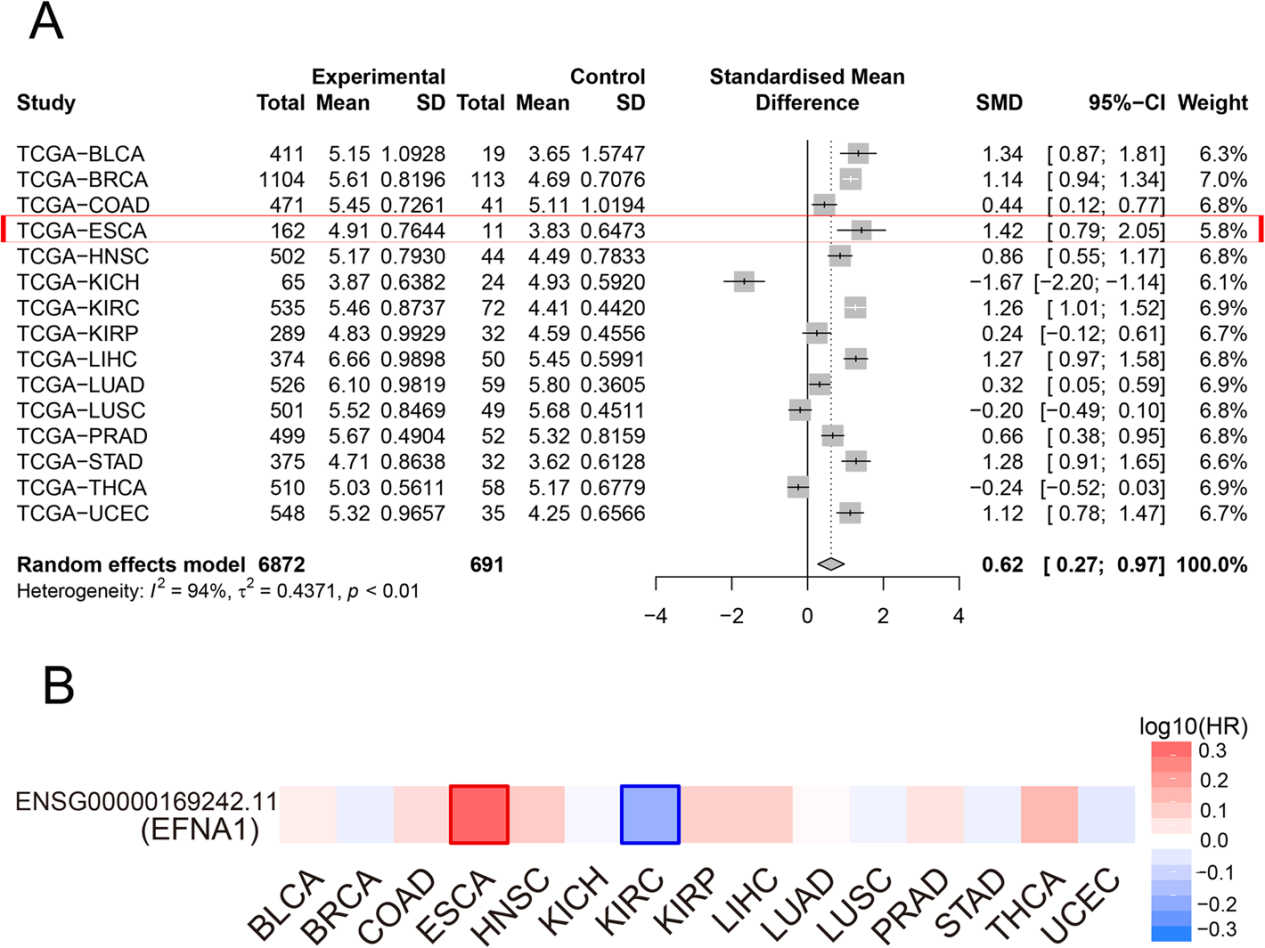
EFNA1 expression in tumor tissue and its clinical significance. **A** Forest map of EFNA1 in 15 common cancers in TCGA. **B** The survival analysis of EFNA1 prognostic impact on multiple tumor patients, which was the most significant in ESCA

To investigate EFNA1 gene expression in tumors, the research group systematically analyzed 15 common tumors in the TCGA database (control samples > 10). In a comparison of 6872 tumors and 691 control tissues, EFNA1 gene was found to overexpress in most tumor tissues, especially in ESCA (Fig. 1A). After further integrating the EFNA1 gene expression and clinical data of 6872 patients, it was also found that the prognosis of patients with tumors was aggravated by EFNA1 gene overexpression, especially in patients with ESCA after screening (Fig. 1B).

### EFNA1 expression in ESCA clinical specimens

**Fig. 2:**
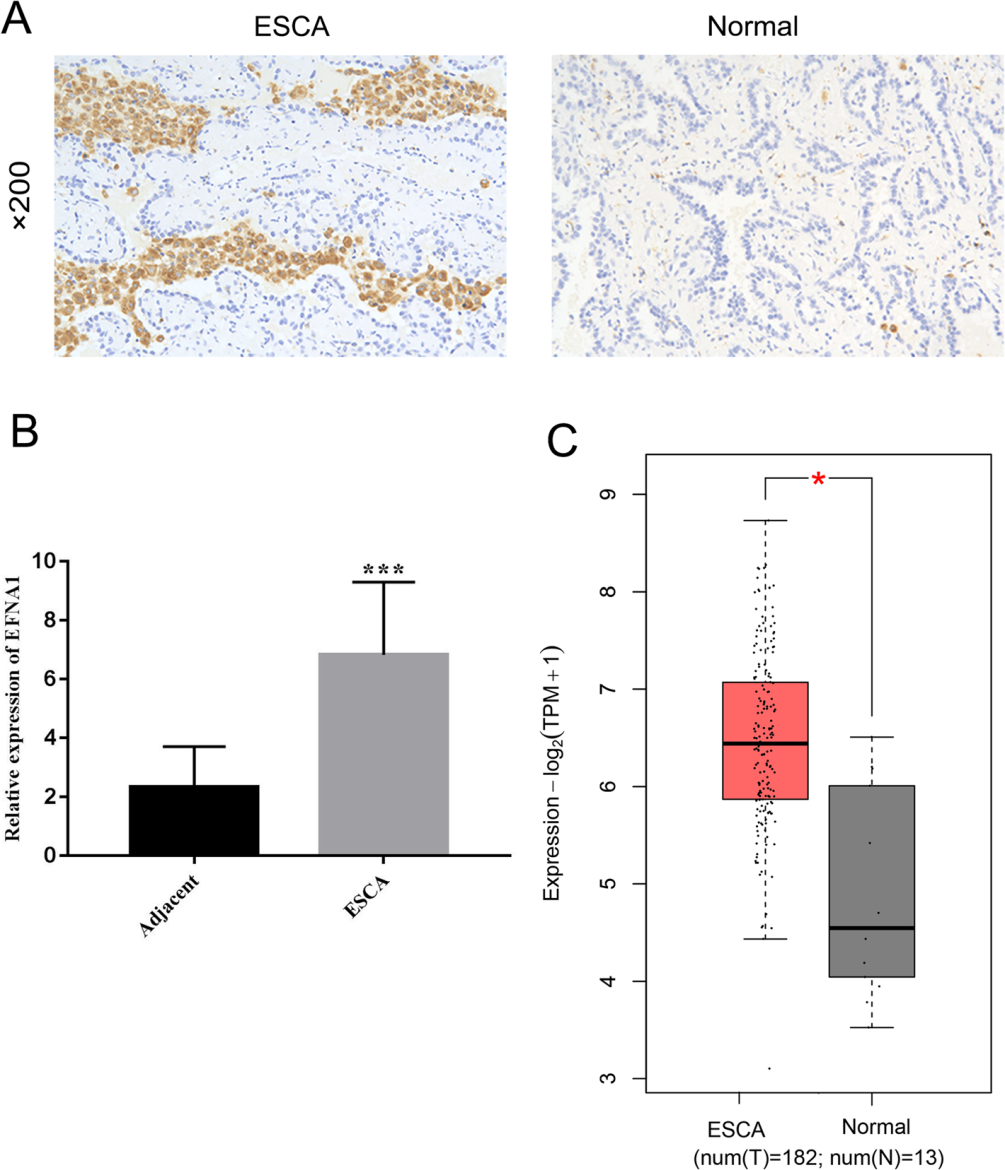
EFNA1 expression in ESCA clinical specimens. **A**, **B** IHC detection of EFNA1 expression in ESCA clinical samples. **C** EFNA1 expression in ESCA and 35 normal tissues, *P < 0.05, **P < 0.01, and ***P < 0.001

In order to verify EFNA1 expression in ESCA tissues, we collected 80 pairs of ESCA tissues and adjacent control tissues from ESCA patients aged 47-79, including 41 males and 39 females. The intensity of EFNA1 expression in the samples was detected with IHC test, showing that EFNA1 expression in cancer tissues was significantly higher than that in adjacent tissues (paired *t* test, *P* < 0.001) (Fig. 2A, B), which was consistent with ESCA in TCGA (182 tumors and 286 normal tissues), and mRNA levels of EFNA1 were significantly higher in cancer tissues than those in normal tissues (*P* < 0.05) (Fig. 2C).

**Fig. 3:**
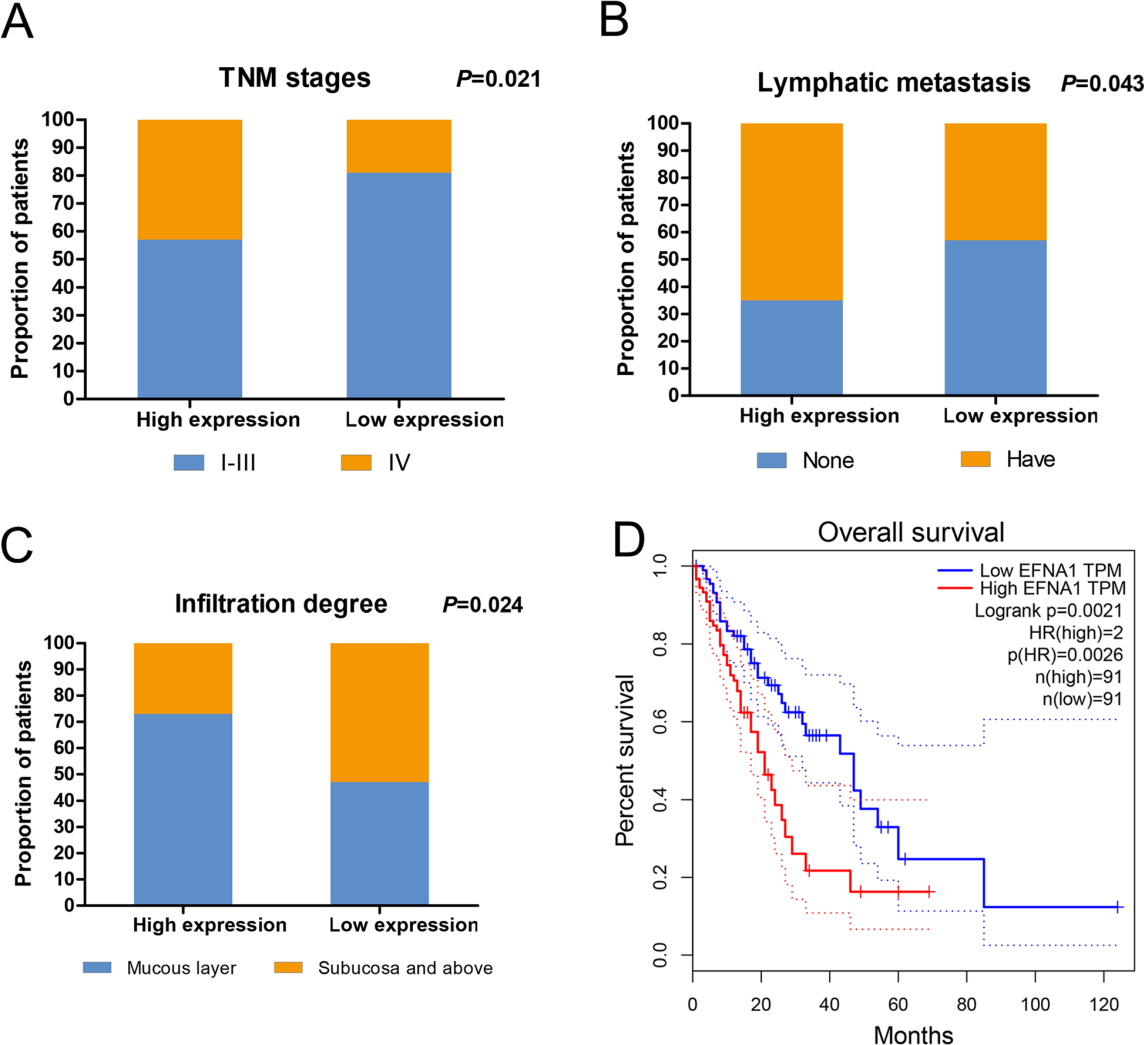
EFNA1 relationship between expression and ESCA clinical parameter characteristics and patient survival. **A**–**C** Relationship between EFNA1 expression and TNM stage, lymph node metastasis, and depth of invasion. **D** In the TCGA dataset, the overall survival time of patients with ESCA was analyzed by Kaplan-Meier method, and it was found that the high EFNA1 expression was related to the poor survival rate of patients with ESCA

Clinical data from patients with ESCA were further collected, to analyze the relationship between EFNA1 expression and ESCA clinical parameters, which showed that EFNA1 expression was associated with TNM staging (*P* = 0.021), lymph node metastasis (*P* = 0.043), and invasion depth (*P* = 0.024) (Fig. 3A–C, supplementary table 1). Kaplan-Meier analysis of the relationship between EFNA1 expression and the prognosis of patients with ESCA showed that the obviously shorter overall survival rate was significantly associated with high EFNA1 expression (*P* = 0.0021) (Fig. 3D). The relationship between survival time and ESCA clinical parameters and EFNA1 expression was further analyzed. In the Cox univariate analysis, age (*P* = 0.037), TNM staging (*P* = 0.031), degree of invasion (*P* = 0.016), and EFNA1 (*P* = 0.011) were associated with the prognosis of patients with ESCA. The results of Cox multivariate analysis showed that only the degree of infiltration (*P* = 0.041) and EFNA1 expression (*P* = 0.022) had independent significance (Table 2). We concluded that EFNA1 protein expression has been confirmed to be predictive to poor survival in patients with ESCA, which might be a good target for esophageal carcinoma therapy.

**Table 2:** Analysis of Cox univariate analysis and multivariate analysis affecting survival time of patients with ESCA

Parameters	Cox univariate analysis			Cox multivariate analysis		
	HR	CI 95 %	*P* value	HR	CI 95 %	*P* value
Age	1.736	1.120–3.214	0.037	–	–	–
Gender	0.798 0	0.291–1.853	0.187	–	–	–
Tumor diameter	0.575	0.258–1.786	0.423	–	–	–
TNM staging	2.035	1.417–3.676	0.031	–	–	–
Lymph node metastasis	0.532	0.346–1.562 =	0.412	–	–	–
Infiltration degree	2.211	1.404–3.782	0.016	1.828	1.142–3.147	0.041
Degree of differentiation	0.657	0.649–1.978	0.215	–	–	–
EFNA1 expression	2.335	1.301–4.926	0.011	2.135	1.236–3.648	0.022

### EFNA1 expression in ESCA cells

**Fig. 4:**
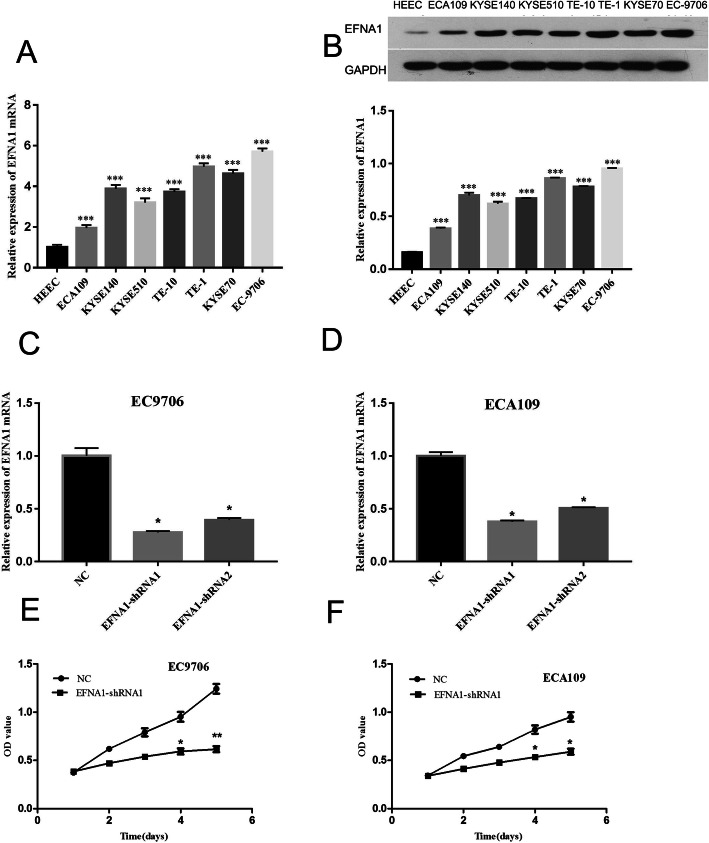
Effects of silencing EFNA1 on ESCA cell proliferation. **A** qRT-PCR test of EFNA1mRNA expression in cell lines. **B** Western blot test of EFNA1 protein expression in cell lines. **C**, **D** After transfection with EFNA1-shRNA, the expression level of EFNA1 was detected by qRT-PCR; NC represented the negative control group. **E**, **F** After EFNA1 was knocked down, the proliferation of ESCA cells changed, and the significance was *P < 0.05, **P < 0.01, and ***P < 0.001

To verify the importance of EFNA1 to tumor cells, 7 ESCA cell lines (EC-9706, ECA109, KYSE140, KYSE510, TE-10, TE-1, KYSE70) and 1 human normal esophageal epithelial cell (HEEC) were selected. The expression of EFNA1 mRNA and proteins was detected with qRT-PCR and western blot methods at the cellular level. The result showed that the expression of EFNA1 mRNA and protein was significantly higher in cancer cells than those in normal cells (*P* < 0.001) (Fig. 4A, B). The expression was the most significant in EC-9706 cell line, and the lowest in ECA109 cell line compared with that of other ESCA cell lines. EC-9706 and ECA109 cell lines were selected for subsequent experiments.

The EFNA1 low-expression ESCA cell model was built with EFNA1-shRNA, and the transfection efficiency was detected with qRT-PCR. The result showed that in EC-9706 and ECA109 cells, EFNA1 expression significantly decreased after EFNA1-shRNA transfection, especially in EFNA1-shRNA 1 group, compared with NC group (*P* < 0.05) (Fig. 4C, D). EFNA1-shRNA 1 model was selected for subsequent experiments.

The findings in the MTT detection showed that, in EC-9706 and ECA109 cells, the cell proliferation was significantly decreased in EFNA1-shRNA1 group compared with NC group (*P* < 0.01, *P* < 0.05) (Fig. 4E, F).

### EFNA1 molecular mechanism affecting ESCA cell proliferation

**Fig. 5:**
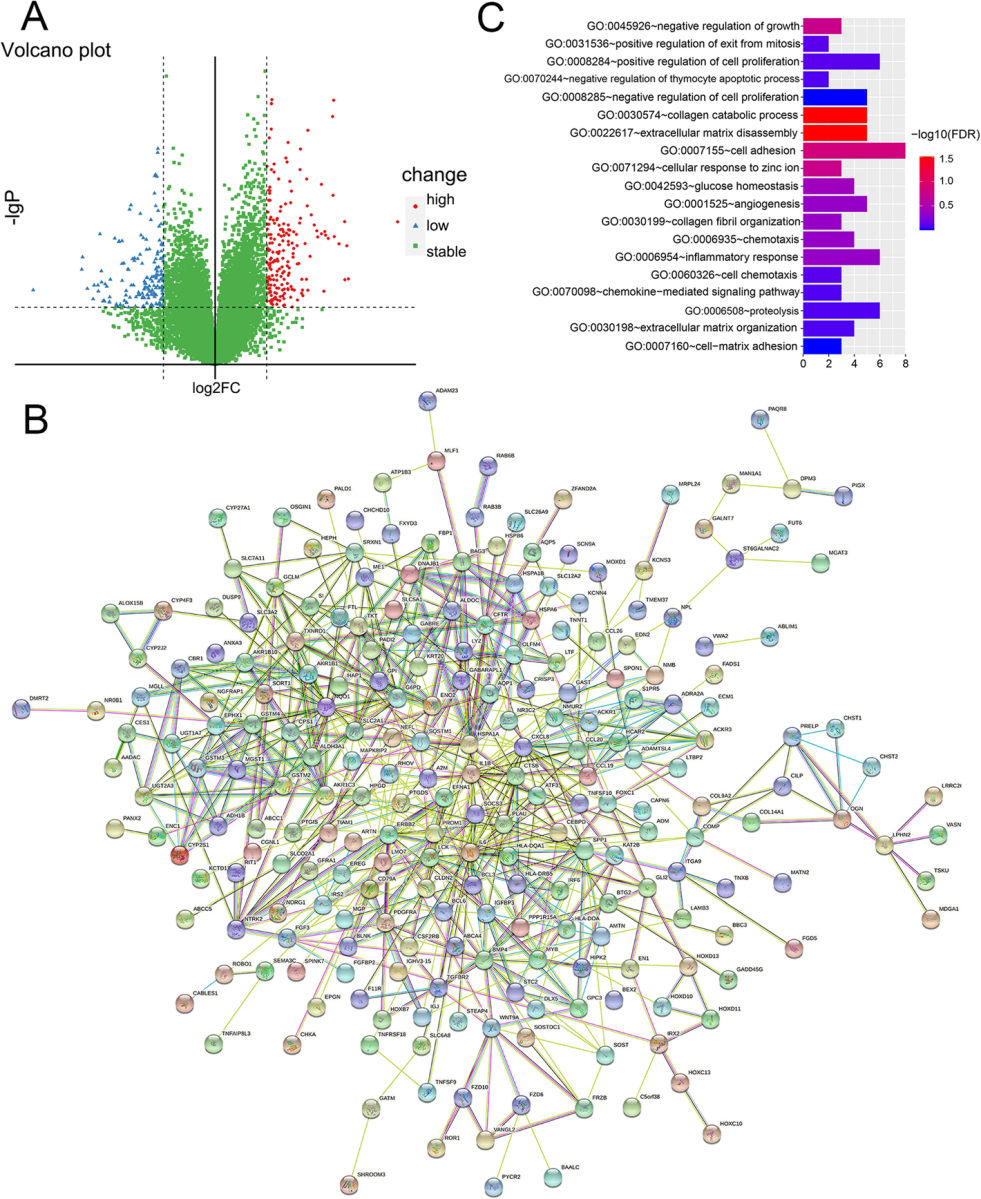
EFNA1 molecular mechanisms affecting ESCA cell proliferation. **A** TCGA data EFNA1 high and low expression of the two groups do differential expression of volcanic map. **B** Network diagram of protein interactions of EFNA1 and 283 different genes (PPI). **C** GO enrichment analysis histogram of EFNA1 and related genes

To explore the molecular mechanism of EFNA1 affecting ESCA cell proliferation, the patient cohort was divided by EFNA1 median expression. A total of 283 differential genes were screened with “edgeR” of the R package as shown in the volcanic map of Fig. 5A, including 192 upregulated and 91 downgraded. Then, the PPI network diagram of EFNA1 related genes was constructed by String (Fig. 5B), and it was found that EFNA1 related genes have complex protein interaction networks. GO enrichment analysis of DAVID pair and EFNA1-related genes showed that the main enrichment pathways were GO0045926: negative growth regulation, GO0031536: positive regulation of mitosis, GO0008284: positive regulation of cell proliferation, etc. (Fig. 5C), which was consistent with the results obtained from the above cell proliferation experiments.

## Discussion

EFNA1 genes are also called B61, ECKLG, EFL1, EPLG1, GMAN, LERK-1, LERK1, and TNFAIP4, which are located on human chromosome 1 and encode EPH family members. Wada et al. studied and reported that EFNA1 can predict the recurrence risk of patients with radical resection of liver cancer, which may become a prognostic marker [22], and some studies have found that it may be related to the prognosis of cervical cancer [23]. Studies have confirmed that EFNA1 may be associated with the prognosis of colorectal cancer, and single nucleotide polymorphism is the main factor leading to genetic susceptibility to tumors. However, Simonian et al. studied and found that there is no significant correlation between the re12904 of polymorphic loci in EFNA1 genes and the susceptibility to colorectal cancer, which may be related to specific populations [24].

Through the analysis of the related data of 15 tumors in the TCGA database, we found that EFNA1 overexpressed in a variety of tumors, especially in ESCA. Further integration analysis indicates that EFNA1 gene overexpression worsened the prognosis of patients with cancers, with the greatest impact on the prognosis of patients with ESCA. Our immunohistochemical results suggest that EFNA1 protein expression in tissues with ESCA is significantly higher than that in adjacent normal tissues, with statistically significant differences (*P* < 0.001). And EFNA1 expression is associated with lymph node metastasis (*P* = 0.043), TNM staging (*P* = 0.021), and degree of invasion (*P* = 0.024), respectively. Tumor invasion is associated with ESCA staging; patients with ESCA with lesions confined to the mucosa have a better prognosis, suggesting that tumors with highly expressed EFNA1 may have a higher malignant biological potential. Excessive proliferation of tumor cells can lead to lymph node metastasis; tumors of upper esophagus are also prone to have lymph node metastasis [25]. This study found that EFNA1 is associated with lymph node metastasis, suggesting that high EFNA1 expression may affect the proliferation and metastasis of ESCA, and early metastasis is also one of the common causes for failure of anticancer therapy in clinical practice and an important factor affecting the prognosis of tumor patients. Thus, upregulation of EFNA1 expression may affect the prognosis of patients with ESCA. Our study showed that EFNA1 protein expression (*P* = 0.011) was significantly associated with shorter survival in patients with ESCA, except for age (*P* = 0.037), TNM staging (*P* = 0.031), and degree of invasion (*P* = 0.016) in univariate analysis. Nevertheless, when COX multivariate analysis was applied, only the degree of infiltration (*P* = 0.041) and EFNA1 protein expression (*P* = 0.022) remained significantly associated with poor overall survival. At present, the main preferred treatment for ESCA is surgery, but postoperative tumor recurrence rate is high. The above results show that EFNA1, as an oncogene, may affect the prognosis of patients with ESCA, suggesting the poor prognosis. EFNA1 may be an effective index to evaluate the prognosis and recurrence of patients with ESCA.

Early detection and progression assessment of esophageal carcinoma require new biomarkers. The early symptoms of ESCA are atypical. Most of the patients with ESCA have progressive dysphagia as the main complaint, which is often the late stage of the disease. EFNA1 is a secreted protein of vascular endothelial cells [26], which highly expresses in ESCA, suggesting that it may be an effective marker for early diagnosis and screening of ESCA.

There is research evidence that EFNA1 is regulatory molecules for many malignant tumors, for example, EFNA1 induces miR-302b expression in malignant mesothelioma (MM) cells and inhibit the growth of tumor spheres by inducing apoptosis [14]. EFNA1 inhibits the proliferation of tumor cells in NSCLC by increasing the expression of tumor suppressor gene cdx-2 [27]. The study of Zhuo et al. found that knocking out EFNA1 can weaken the invasiveness of gastric cancer cells. In vivo experiments have confirmed that knocking out EFNA1 can weaken the distant metastasis ability of gastric cancer and provide a target for targeted therapy of gastric cancer [28]. Yamamoto et al. studied and found that EFNA1 may be an effective index to evaluate the prognosis of colorectal cancer, and their cell experiments have also confirmed that knocking down EFNA1 can inhibit tumor proliferation, migration and invasion [29]. The study by Kandouz et al. also confirmed that the EFN family may reduce the adhesion between cells and lead to metastasis of tumor cells [30]. In this study, 8 ESCA-related cell lines were selected. And EFNA1 expression was detected with qRT-PCR and WB at the ESCA cell level. The results showed that the expression of EFNA1 mRNA and proteins was significantly higher in cancer cells than that of normal cells. EFNA1 can highly express in ESCA cells and may play the role of oncogenes. EC-9706 cells with the highest EFNA1 expression and ECA109 cells with low EFNA1 expression were transfected with EFNA1-shRNA1 and EFNA1-shRNA2 to construct a cell model with low EFNA1 expression. It was found that EFNA1-shRNA1 group had a particularly significant decrease in EFNA1 expression, so EFNA1-shRNA1 was selected for subsequent experiments. Proliferation experiments were performed in EC-9706 and ECA109 cells transfected with EFNA1-shRNA1, and the results showed that knocking down EFNA1 can inhibit the proliferation of ESCA cells.

## Conclusions

To sum up, this study found that EFNA1 expression in ESCA tissues is significantly high, which is related to the prognosis of ESCA. It may be an independent risk factor affecting its prognosis, which provides an important basis for clinical evaluation of the prognosis of patients with ESCA. Knocking down EFNA1 can affect the biological process of ESCA cell proliferation at the cell level and is expected to be a new target for ESCA therapy. However, there are limitations in this study. There is no large sample research and no animal experiments. These problems provide directions for future research.

## Supplementary Information

**Additional file 1.** Nomogram.

**Additional file 2.** EFNA1 Relationship between expression and ESCA characteristics of clinical parameters.

## Data Availability

The datasets used and/or analyzed during the present study are available from the corresponding author on reasonable request.
