# Supplementary material for: Effects of EFNA1 on cell phenotype and prognosis of esophageal carcinoma
Source: World J Surg Oncol. 2021 Aug 16;19:242. doi: 10.1186/s12957-021-02362-8 (PMC8369630; doi:10.1186/s12957-021-02362-8)
Supplement: Supplementary file 1 — Additional file 1. Nomogram. [file 12957_2021_2362_MOESM1_ESM.pdf]

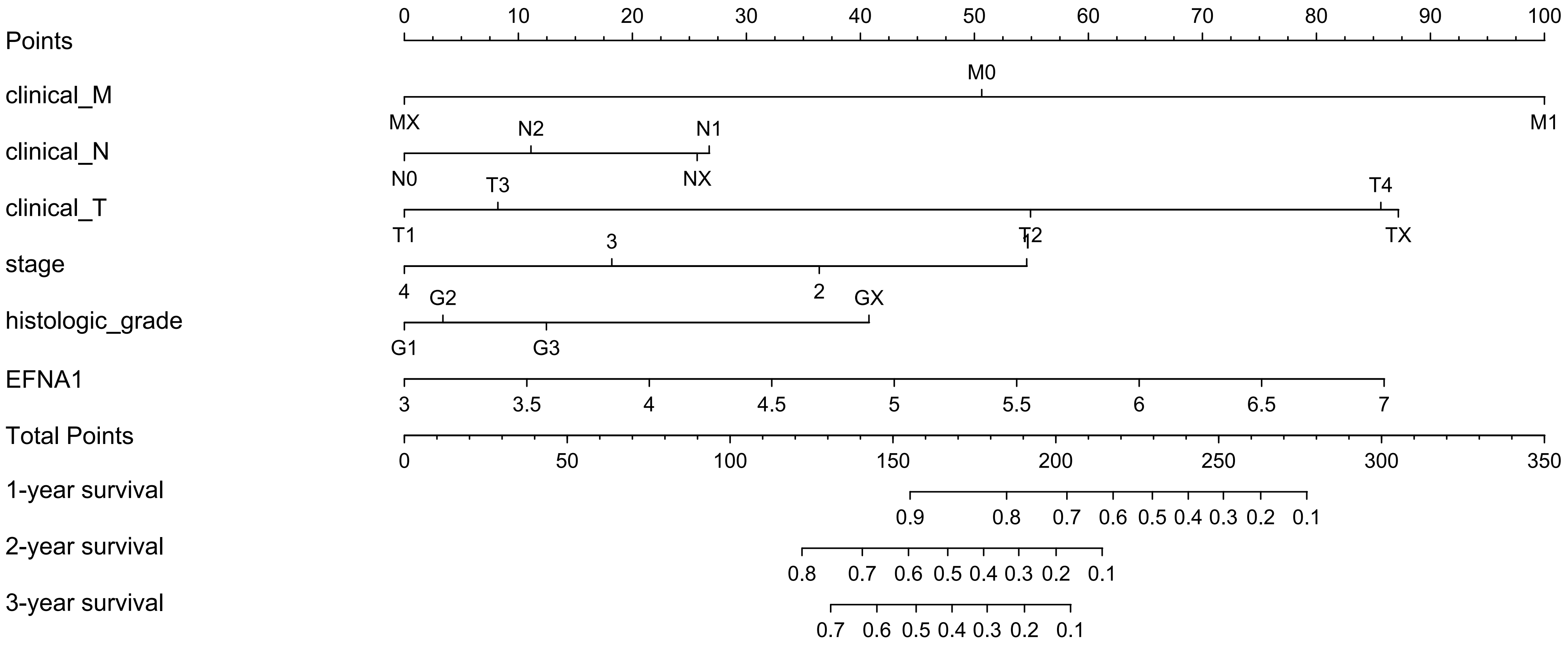
Points
0 10 20 30 40 50 60 70 80 90 100
clinical_M
M0
MX
M1
clinical_N
N2 N1
N0 NX
clinical_T
T3 T4
T1 T2 TX
stage
3 1
4 2
histologic_grade
G2 GX
G1 G3
EFNA1
3 3.5 4 4.5 5 5.5 6 6.5 7
Total Points
0 50 100 150 200 250 300 350
1-year survival
0.9 0.8 0.7 0.6 0.5 0.4 0.3 0.2 0.1
2-year survival
0.8 0.7 0.6 0.5 0.4 0.3 0.2 0.1
3-year survival
0.7 0.6 0.5 0.4 0.3 0.2 0.1
