# Supplementary material for: Effects of EFNA1 on cell phenotype and prognosis of esophageal carcinoma
Source: World J Surg Oncol. 2021 Aug 16;19:242. doi: 10.1186/s12957-021-02362-8 (PMC8369630; doi:10.1186/s12957-021-02362-8)
Supplement: Supplementary file 2 — Additional file 2. EFNA1 Relationship between expression and ESCA characteristics of clinical parameters. [file 12957_2021_2362_MOESM2_ESM.docx]

**Table I EFNA1 Relationship between expression and ESCA characteristics of clinical parameters**

| Parameters |  | N | EFNA1 expression | | Χ^2^ | P |
| --- | --- | --- | --- | --- | --- | --- |
|  |  |  | High | Low |  |  |
| Age (years) | ≥60 | 46 | 26 | 20 | 0.222 | 0.638 |
|  | <60 | 34 | 21 | 13 |  |  |
| Gender | Male | 41 | 25 | 16 | 3.184 | 0.074 |
|  | Female | 39 | 16 | 23 |  |  |
| Tumor diameter (cm) | <5 | 48 | 27 | 21 | 0.302 | 0.583 |
|  | ≥5 | 32 | 16 | 16 |  |  |
| TNM staging | Stage I-III | 57 | 21 | 36 | 5.331 | 0.021* |
|  | Stage IV | 23 | 15 | 8 |  |  |
| Lymph node metastasis | No | 35 | 13 | 22 | 4.114 | 0.043* |
|  | Yes | 45 | 27 | 18 |  |  |
| Infiltration degree | Mucosal layer | 29 | 10 | 19 | 5.119 | 0.024* |
|  | Submucosa and above | 51 | 31 | 20 |  |  |
| Degree of differentiation | Medium High Differentiation | 44 | 25 | 19 | 0.013 | 0.910 |
|  | Low differentiation | 36 | 20 | 16 |  |  |

Note :* represents P <0.05.
